# The Helping Hand in Ukraine: feasibility and potential impact

**DOI:** 10.1186/s40814-024-01520-5

**Published:** 2024-06-29

**Authors:** Solfrid Raknes, Tetiana Chorna

**Affiliations:** 1 Molde University College, 6410 Molde, Norway; 2 Heks-Eper Ukraine, Odesa, Ukraine

## Abstract

**Background:**

New services are needed to prevent the mental health consequences of the war in Ukraine. Ten adolescents self-recruited to use and evaluate the Ukrainian version of the Helping Hand (HH) in Odesa, Ukraine. From April to June 2023, they participated in a 10-session group program where they played the cognitive behavioral game app, shared stories, and engaged in activities to enhance their coping skills.

**Methods:**

A mixed-method, quantitative–qualitative design was used to get insight into the feasibility and potential impact of the HH on Ukrainian adolescents’ mental health and well-being during the war. A questionnaire to the adolescents assessed the feasibility of the intervention; anxiety and depression symptoms were assessed before and after the intervention by a standardized and validated adolescent-completed questionnaire. An interview with the psychologist who implemented the intervention was used to interpret the data completed by the adolescents.

**Results:**

Eight of 10 adolescents completed the HH intervention, and the psychologist found the HH helpful and culturally appropriate. The average anxiety and depression symptoms decreased from before the intervention (*M* = 20.4) to after (*M* = 15.0), showing a moderate effect size.

**Conclusion:**

The results indicated that the HH has a high potential to prevent mental health consequences in Ukraine.

## Key messages regarding the feasibility

- What uncertainties existed regarding the feasibility? Before the study, it was unclear whether the HH program, translated to Ukrainian, would be accepted and found relevant by adolescents and mental health specialists in the context of Ukraine’s ongoing war.
- What are the key feasibility findings? The adolescents and mental health specialists warmly welcomed the HH program. The content was culturally relevant and appropriate, the language resonated well with the adolescents, and the blended learning program seemed easy to implement given the already established digital skills and available digital tools among adolescents and mental health specialists.
- What are the implications of the feasibility findings for the design of the main study? Future research on the HH for Ukrainian adolescents should use larger and controlled samples, assess impact at several measure points with validated scales, use independent evaluators, and explore scalability and comparative effectiveness across various implementation settings.

## Introduction

War brings about various adversities, such as witnessing violence, experiencing displacement, losing loved ones, and dealing with uncertainty at many levels, challenges known and discussed by mental health professionals involved in the war in Ukraine [1]. Such adversities can overwhelm adolescents who experience war and make it difficult to process emotions and develop healthily [2]. Anxiety, depression, traumatic memories, PTSD, suicides, mistrust in people, and lack of hope for the future are associated with war experiencing war [3]. Grief and trauma can leave war-exposed adolescents wordless about what they struggle with most. Instead of sharing what is on their mind, the intense feelings can create a barrier to sharing [4].

Prevention programs targeting adolescents are recommended to reduce the burden of mental health disorders [5, 6]. Social and emotional interventions can support adolescents to cope better with stress, anxiety, and grief [7, 8]. Arenas for sharing feelings and thoughts and learning where to seek support if needed can be helpful for adolescents growing up in all types of circumstances and crucial for adolescent mental health and overall resilience during wartime [9].

Adolescents may experience a wide range of intense emotions during war, including fear, anger, sadness, or confusion. Exploring and expressing emotions openly can be therapeutic for adolescents [10, 11]. By encouraging adolescents to talk about their feelings, they can better understand their emotions and find validation and support from others experiencing similar struggles [12]. Sharing experiences and emotions can help reduce feelings of isolation and foster a sense of belonging and connectedness [13]. Further, learning the basic cognitive behavioral techniques, such as problem-solving skills and awareness of emotions and thoughts, seems to prevent mental health disorders [14].

Programs to increase coping in adolescents can take many forms, both in person and digitally [15]. Studies have shown that game-based learning interventions offer the potential for increasing engagement and motivation, which have natural ties to learning [16, 17]. A recent review suggests that digital teaching aids are most effective when they include some form of human interaction, such as a collaboration between game users or guidance from a facilitator [18]. Blended learning is an education style in which students learn from digital tools combined with traditional face-to-face teaching.

The Helping Hand (HH) is a digital game for adolescents, a cognitive behavioral tool (CBT) created to reduce the burden of mental health problems across cultural and economic divides [19]. The game forms the base for the HH program, a blended learning psychosocial support program for adolescents 12–18 years. How to run the HH program is described session by session in a detailed manual for group facilitators [20]. Pilot and feasibility studies of the HH among Syrian refugees in Lebanon have found that adolescents found the game easy to use, and having played the game was associated with higher well-being and lower anxiety and depression when implemented in groups of adolescents [21–24]. The analog version of the HH was proven to reduce anxiety and depression when implemented in groups of adolescents as a school-based program in Norway [25], as well as standard CBT based on 1-year follow-up data [26]. Also, the HH game won the UpLink — World Economic Forum´s Youth Mental Health Challenge 2022.

Modern health care aims to use evidence-based interventions [27]. Culture and context are supposed to be crucial when the aim is to support adolescents affected by war and central when considering the feasibility of a psychosocial intervention [2]. Appropriate focus areas when assessing feasibility are acceptability, demand, implementation, practicality, adaptation, integration, and expansion [28]. However, the potential impact of an intervention depends not only on the program and how it is implemented but also on when it is implemented and with whom [29].

### Objectives

The study aimed to evaluate the feasibility and potential impact of the HH program among adolescents in Ukraine. The research questions focused on the acceptability of the HH intervention among adolescents and psychosocial support (PSS) staff, the need for cultural adaptations of the HH game and manual to a Ukrainian version of the program, and the potential impact of the HH program in Ukraine during the war.

## Methods

### Research design

A mixed-method, quantitative–qualitative, one-group, pre-post design was used.

### Beneficiaries and context

The Helping Hand project was implemented in Odesa, a city by the Black Sea in southern Ukraine, by the nongovernmental organization (NGO) HEKS-EPER Ukraine. The recruitment was conducted through information on the NGO’s website, which specified that participants should be within this age range and residing in Odessa. This recruitment procedure followed the NGO’s standard procedure for recruiting adolescents to their psychosocial services. Ten adolescents aged between 13 and 17 years (*M* = 15.5; *SD* = 1.32) were self-recruited to participate in the study. No exclusion criteria were applied, and all adolescents in the given age group were welcome to participate. This method aimed to target adolescents directly affected by the ongoing conflict, ensuring the relevance and immediacy of the intervention’s impact on this demographic. Their parents provided informed consent for their children’s participation in the program, which included an evaluation component, and the adolescents provided their consent.

The study was conducted from April 12 to June 14, 2023. Weekly 90-min sessions were held during this period, which was marked by increasing wartime activities, including regular missile and drone attacks on Odessa. Notably, on June 14, 2023, the day post-test data was collected, a bombing occurred in Odessa, resulting in casualties and injuries. Despite these challenging circumstances, daily life and annual school exams continued for the adolescents, adding layers of stress.

### Intervention

**Fig. 1:**
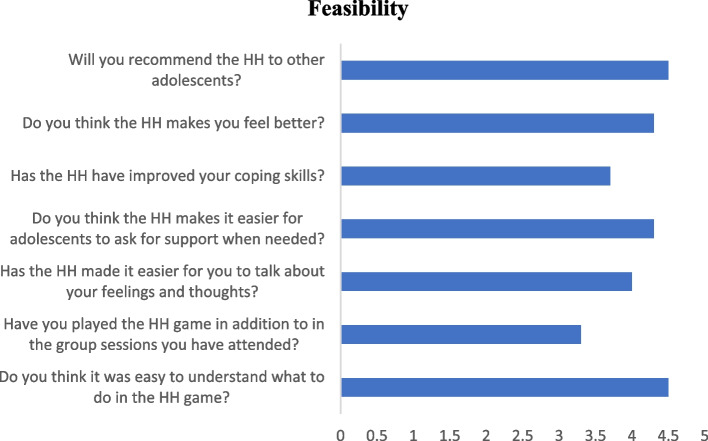
The Helping Hand problem-solving system. Giving an overview of how the adolescents rated the feasibility of the Helping Hand. Numbers are reported as mean scores on each item on a scale from 1 (not at all) to 5 (yes, very much)

The HH PSS program is a 10-session blended learning group, a cognitive behavioral intervention. The overall learning goals of the program are to (a) raise awareness of emotions and the way of sharing them; (b) raise awareness of relationships between situations, feelings, thoughts, and behavior; (c) raise awareness of automatized thinking and to learn about negative automatic thoughts (*red thoughts*) and helpful thoughts (*green thoughts*); (d) improve coping strategies and problem-solving skills for use in emotionally challenging situations by learning the Helping Hand problem-solving system (see Fig. 1); and (e) improving social skills, communication skills included.

The HH game was translated into Ukrainian by a professional translation company, and all voices used in this version of the app are Ukrainian. A detailed session-to-session manual for guiding the group facilitator in running the program was translated into Ukrainian from English for this implementation and by a professional translation service. No other cultural adaptations were made in the manual or the game itself. Table 1 gives an overview of the themes and the specific learning goals of the HH program. The psychologist who implemented the program participated in a 5-h HH digital training workshop for PSS staff before implementing it.

**Table 1:** Overview of the Helping Hand program

Session	Theme	Learning goals
1	Presentation anxiety	Learn that fear of presentations can be reduced by practice, and that making mistakes is a necessary part of improving presentation skills
2	Dealing with criticism	Learn how to give and receive criticism in ways that facilitate learning and strengthen relationships
3	Rejection	Increase awareness on your inner dialogue and how we can talk ourselves up and down. Strengthen skills to initiate and develop healthy relationships
4	Depressed mother	Increase health literacy and decrease stigma about depression Learn to identify pessimistic and optimistic thought patterns. Learn that unhelpful thoughts can be questioned, and that destructive thought patterns can be challenged
5	Heartbroken	Increase awareness of social support and active coping strategies when suffering from painful loss
6	Beautiful?	Identify what you like with yourself and others and get experiences in talking about that. Practice giving and receiving compliments
7	Bullying and racism	Empower adolescents to stand up against bullying, harassment, and racism
8	Suicidal thoughts	Increase awareness of the need for support when suicidal thoughts appear and practice how to support friends in very hard times
9	Bad memories	Increase awareness of how traumatic memories can create hardship, and learn that a non-judging attitude towards emotions can make it easier to live in peace with the past
10	Daring to tell your opinion	Learn about group pressure and reflect on the value of standing up for what you think is right

The Helping Hand program consists of 10 sessions. Each of the sessions has specific learning goals and is accompanied with one scenario in the Helping Hand digital game where the task is to support the main character to cope with a theme-related challenge

### Institutional review approval and data security

This research was maintained per HEKS-EPER Ukraine’s guidelines and privacy standards. The data collected are stored securely in their storage facilities for health data to avoid potential theft and misuse [30]. Furthermore, all data collection and storage connected with this research were in line with the General Data Protection Regulation (GDPR), and no data about the user is stored in the game itself. In the study, ID numbers were created and used to connect digitalized health data from the same individual at different data points. All digitalized health data was anonymized, and the list of names that combined the individual participants with their ID numbers was made and stored safely and in analog only.

### Evaluation of outcome

To measure the potential impact of the intervention, the adolescents completed paper-and-pen-administered questionnaires before and right after the intervention. Anxiety and depression symptoms were collected before and after the intervention; well-being was assessed before the intervention only, and feasibility was evaluated after the intervention. Interpretation of the data was completed with a critical informant interview with the psychologist who implemented the program and who is also the second author of this paper.

### Measures

*A feasibility questionnaire* was made by the first author for this study and included seven quantitative items, assessed on a scale from 1 (not at all) to 5 (yes, very much). Examples of the items are “Do you think it was easy to understand what to do in the HH game?” “Has the HH made it easier for you to talk about your feelings and thoughts?” “Has the HH improved your coping skills?” and “Will you recommend the HH to other adolescents?” The questionnaire included one open-ended question: “What did you learn from the HH?”.

*The Revised Children’s Anxiety and Depression Scale* (RCADS) is a 25-item validated questionnaire [31] that has been widely used to assess anxiety and depression symptoms in children and adolescents and is free to access [32]. The general psychometric properties of the RCADS-25, such as its internal consistency, test–retest reliability, and convergent validity, have been found robust in diverse populations, suggesting its applicability in different settings [33, 34]. On the RCADS-25, each item is scored as never (0), sometimes (1), often (2), and always (3). A score of 27 or higher on RCADS was used for screening for anxiety and depression. Examples of statements are “I feel sad or empty,” “I feel afraid of being at home alone,” “I have trouble sleeping,” and “I have no energy for things.” We used the Ukrainian version the Inter-Agency Standing Committee recommended for evaluations in fragile contexts in 2021 [35]. However, we did not find validation studies of RCADS-25 conducted in Ukraine or Ukrainian, nor specific norms for Ukrainian youths.

*The World Health Organization’s 5-item Wellbeing Index (WHO-5)* is a 5-item questionnaire scored from 0 (at no time) to 5 (all the time), and a raw scoFre below 13 was used to indicate poor well-being. The sum score is multiplied by 4 to get a scale from 0 to 100, with higher scores indicating better well-being [36]. Examples of statements in WHO-5 are “I have felt cheerful and in good spirits” and “My daily life has been filled with things that interest me.” However, we did not find validation studies of WHO-25 conducted in Ukraine nor a description of the translation process into Ukrainian.

## Results

### Participants

The sample size for this study was determined based on the NGO’s intention to pilot the intervention with one group initially. The NGO decided the group size should not exceed 10 participating adolescents to ensure manageability and effective intervention delivery by one group facilitator. The targeted number of participants was met without difficulty, indicating strong interest and suitability of the intervention for the target population. The participants were between 13 and 17 years old (*M* = 15.5; *SD* = 1.32). One of the recruited adolescents never turned up, and one stopped coming after the second session. Eight adolescents completed the entire intervention. The participant who dropped outscored in the normal range of anxiety/depression symptoms at the pretest (21 on RCADS) but reported low well-being (score four on WHO-5). The reason for the dropout was unknown, and no post-data was collected from this participant. Wellbeing assessed by WHO-5 before the intervention in this sample (*N* = 9) was relatively low (*M* = 56; *SD* = 24.85). Boys’ wellbeing was slightly higher (*M* = 56) than girls (*M* = 51). Average levels of anxiety and depression as assessed by RCADS were within the normal range (*M* = 20.4; *SD* = 14.39) and ranged from 13–52, without significant gender differences (boys, *M* = 22.3; girls, *M* = 22.9). Two adolescents reported high scores of anxiety/depression symptoms (*RCADS* ≥ 27) and low well-being (WHO-5 < 13) at baseline.

### A feasible intervention for Ukrainian adolescents

The adolescents completed the feasibility survey after the last intervention session, and the questions were rated on a scale from 1 (not at all) to 5 (yes, very much). As shown in Fig. 1, the adolescents (*n* = 8) found it easy to understand what to do in the HH game (*M* = 4.5), indicating a high level of clarity. They also reported that the game made it easier to express their feelings and thoughts (*M* = 4). Moreover, they noted that they thought the game made seeking support easier when needed (*M* = 4.3). They thought the game positively impacted their well-being (*M* = 4.3). Finally, the adolescents recommended the HH game to other adolescents (*M* = 4.5).

There was a variation among the participants in their reporting on whether they thought their coping skills were improved in the intervention period. At the same time, six adolescents reported high positive scores of 4 or 5 on this item; one reported no improvement, as indicated by a score of 1. One scored 3, indicating uncertainty regarding the improvement of coping skills. When it comes to use of three of the eight, adolescents had been playing the game a lot at home in addition to when they met in the group; one had played it a little at home, and half of them had not played it at home at all.

When asked what they had learned from the HH, the adolescents answered:

“I have learned a lot.”

“Learned to talk to people and calm down”

“Greater understanding of how to behave in different situations”

“Self-love”

“Identify emotions and openly talk about my feelings.”

“Deeper understanding of different situations”

The critical informant interview with the psychologist described the program as successful. During the first sessions, the adolescents gradually developed trust in each other in the group. They liked the game, and the psychologist could see how they related to the content in all sessions. Session 4, “Mom is depressed,” was highlighted as particularly relevant, as families are split due to the war, and many of the adolescents are living with their mothers only. The older adolescents typically are given much responsibility, and playing a scenario where they helped the main character deal with a mother’s depression made them start sharing lots of stories. The psychologist recommended the program as important and relevant at the national level. Some minor language mistakes in the game were reported and should be corrected.

### Levels of anxiety and depression

**Fig. 2:**
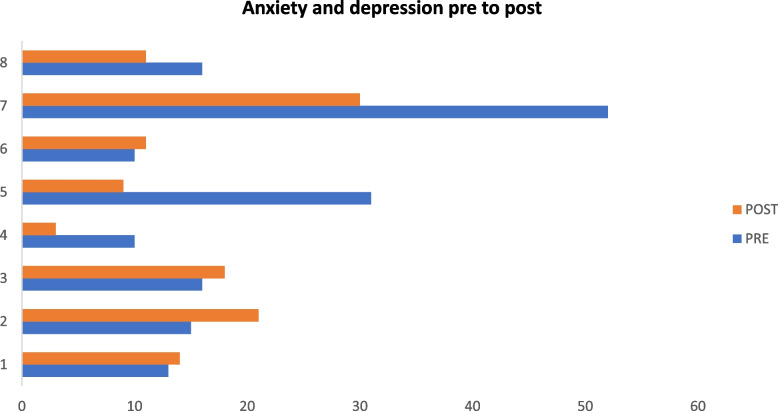
Showing how the level of anxiety and depression changed from pretest to posttest in each of the eight adolescents, as assessed with the Revised Children’s Anxiety and Depression Scale (RCADS) before and after the intervention. Numbers on the *Y*-axis refer to each participant’s ID number, and numbers on the *X*-axis refer to the RCADS score on a scale; higher scores represent higher symptoms

The average anxiety and depression symptoms in adolescents who completed the intervention (*n* = 8) decreased from before the intervention (*M* = 20.4; *SD* = 14.39) to after (*M* = 15.0; *SD* = 8.29). The effect size, calculated using Cohen’s d, was 0.46. Figure 2 shows how the level of anxiety and depression changed from pre to post for each of the eight participants who completed the program. Notably, two of the adolescents who reported high levels of symptoms before the program started (ID numbers 5 and 7) reported far lower symptoms after the program than before.

## Discussion and implications

This study provides insights into the feasibility and potential impact of the HH game for Ukrainian adolescents living in a war zone. The study found that the HH program was feasible and showed promise in reducing anxiety and depression symptoms.

### The HH was well-accepted and relevant

The adolescents reported that the game made it easier to talk about feelings and thoughts and cope with challenges and was relevant for adolescents growing up in a war zone. When exposed to violence and living with high risks over a prolonged period, acquiring a broad range of emotional regulation skills that can enable coping with stress, insecurity, grief, anxiety, and bad memories is essential [15]. “Empowering the adolescents to rely on themselves and their inner resources is central for what´s needed for adolescents growing up in Ukraine in this situation,” the psychologist argued, highlighting this need as she experienced it in Odesa. Interventions that make it easier for adolescents to share their feelings and thoughts and to cope with challenges are successful at the core of psychosocial support programs [7, 37].

Further, the adolescents also reported that the HH made it easier to reach out for help when needed, indicating less stigma associated with help-seeking behaviors. As the stigma associated with help-seeking is reported to be a central barrier for people affected by war [38], this is an important finding, strengthening the potential impact of a digital mental health game. Further, since adolescents in war-affected areas typically face significant disruptions to their social support systems [39], the skill of help-seeking is essential. When families are separated, whole communities displaced, and traditional support structures weakened, being able to seek support from trustworthy, available friends, family members, teachers, mental health professionals, or others becomes more critical than under more stable, regular, and supportive circumstances. Supportive relationships can increase resilience [40], providing a safe space for adolescents to share their experiences, receive guidance, and access resources to address their emotional needs. Healthy relationships and good coping skills can help adolescents restore a sense of normalcy amidst the chaos of war [41].

### Empowerment in a post-Soviet Union state

The fact that the psychologist said the game encouraged communication and support-seeking behaviors among adolescents is relevant in this setting. For adolescents in a post-Soviet-union state, training in expressing their views and sharing what is on their minds might be crucial for how their society develops [42]. Good experiences with sharing feelings and thoughts might increase a sense of freedom that can be empowering [7]. Further, actual access to a broad range of adolescent mental health services depends on adolescents who actively want and dare to participate. Likewise, active participation in the community relies on civilians’ daring to raise their voices; witnesses from various points of view are the basis for democracy in its broadest sense.

Both recruitment and retention in this study followed the NGO’s standard procedures for psychosocial services. They usually recruited adolescents to their services through their web page, implemented sessions in their center, and sent text messages to the adolescents and their parents reminding them about the sessions. The intervention was implemented as suggested by the first author, using a group-based, blended learning approach with 10 sessions and pre- and post-assessments. All adolescents, parents, and the health professionals involved had access to smartphones, electricity, and Wi-Fi, which was available in Odessa most of the time during the implementation period. Notably, the adolescents had good enough digital skills to use the game, and the psychologist had high enough digital pedagogical skills to embrace the concept of blended learning without extra learning time, witnessing a population with high digital competency and access to modern digital tools.

### Symptom decrease

Our findings showed a notable decrease in average anxiety and depression symptoms, from a mean score of 20.4 (*SD* = 14.39) before the intervention to 15.0 (*SD* = 8.29) after the intervention. This moderate effect size suggests that the program has a meaningful impact on mental health outcomes for adolescents with symptoms of anxiety and depression. The decrease is lower than the HH implemented among Syrian refugees in Lebanon (Raknes et al.: Expanding access to mental health: evaluation of a severe mental health game for adolescents, submitted). Notably, the two participants who initially reported high symptoms experienced a significant reduction in these symptoms post-intervention. The adolescents reported that they thought the HH game made them feel better; this links the decrease in anxiety and depression with the HH program. Further, the psychologists’ interpretation of observed changes in adolescents during the implementation period also highlights the intervention as impactful. The outcomes of this study align well with research on adolescents to promote healthy development and prevent mental health disorders [43].

### Scalability

Whether a mental health intervention is successfully implemented depends on critical factors, where aspects of the intervention are one central part [44]. Also, the fact that the HH program is an e-health program can increase its scalability [45]. The adolescents in this study reported that the game’s instructions and mechanics were clear and easy to comprehend, factors associated with successful implementation [46]. Moreover, the high willingness of participants to recommend the HH game to other adolescents reflects the game’s perceived value and positive reception among the participants. Further, the health professionals seemed to need more support to understand how to use the digital game and manual, witnessing high digital competence and demonstrating strong skills in teaching blended learning and digital pedagogical methods. In this study, the group facilitator learned how to run the HH program with adolescents from a digital, group-based 5-h workshop. The fact that the HH intervention seemed quick and easy to understand and could be implemented by teachers, social workers, other professionals, and psychologists was assessed as necessary to expand the program in Ukraine, the local psychologist highlighted. The number of well-educated, specialized mental health professionals is low in Ukraine, and new models for psychosocial support are needed [47]. CBT tools implemented by nonspecialists are a basis for many scalable and cost-efficient evidence-based interventions [48]. Conversely, a decade of digital interventions has also revealed that underserved groups do not always benefit from such interventions [49].

Further, researching this war zone taught us several valuable lessons for wider-scale evaluations. Our key strategies included early engagement with the local community, flexible and adaptable methods building on the services provided by the implementer, cultural sensitivity was a clear intention during the training and supervision of the mental health professionals, and practical and emotional support at all levels in the implementing organization was offered. We tried to role model strict ethical standards.

### Limitations

While the findings from this study were promising, it is essential to acknowledge the limitations. The small and self-recruited sample, the absence of a control group, and pro-post design limit the generalizability of the results. Also, a possibility of confirmation bias is present, as the innovator of the HH app took a central part in the research through training, supervision, facilitation of the study, and analysis of the data. These are typical limitations of small feasibility [28]. Further, even if we used standard questionnaires 29 and 30 (RCADS-25 and WHO-5) recommended for use with adolescents in fragile settings by reputable institutions, we did not find validation studies of these instruments conducted in Ukraine or Ukrainian, no specific norms for Ukrainian youths, and no description of the translation process. In a potential upscaled study, the translation process should be validated. On the other hand, the mixed-methods approach allowed for methodological triangulation, which enhanced the scope and rigor of our study, providing a more in-depth understanding of the acceptability, usefulness, and potential impact of the intervention [50], and a pragmatic approach made this study possible. The study results encourage applying more robust methods, and a larger sample was recruited from various implementation settings in further studies of the HH program.

## Conclusion

The HH program combines the stimulation of mental health literacy with the teaching of social and emotional skills and, at the same time, serves as an early treatment intervention for adolescents with anxiety and depression. This approach seems particularly relevant in the context of the war in Ukraine, where low mental health literacy and stigma hinder help-seeking, and adolescents are growing up with an increased risk of developing mental health disorders. Further, the e-health format is a strength of the HH program. It can be crucial for access to psychosocial support in a conflict zone where traditional mental health services may be lacking or inaccessible. Combining the group format where adolescents meet in person with a digital mental health game, as done here, is probably more impactful than the game when used as a self-help tool only [51].

Overall, the findings suggest that the HH game has the potential to prevent mental health disorders in a Ukrainian setting by enhancing adolescents’ coping and communication skills, enhancing support-seeking behaviors, and decreasing anxiety and depression in adolescents struggling with these problems. Our results contribute to the accumulating evidence on digital health interventions in various settings [52]. Building peace is essential to promote mental health [53]. Simultaneously, for adolescents growing up in war, access to evidence-based, culturally adapted, and acceptable psychosocial support interventions is urgently needed [37]. Future studies should explore these preliminary findings across a wider demographic and use more rigorous research methods and larger samples to learn more about the impact of the intervention.

## Data Availability

All data are available on request.
